# Specific anti-glycan antibodies are sustained during and after parasite clearance in *Schistosoma japonicum*-infected rhesus macaques

**DOI:** 10.1371/journal.pntd.0005339

**Published:** 2017-02-02

**Authors:** Y. Y. Michelle Yang, Xiao Hong Li, Katarzyna Brzezicka, Niels-Christian Reichardt, R. Alan Wilson, Angela van Diepen, Cornelis H. Hokke

**Affiliations:** 1 Department of Parasitology, Leiden University Medical Center, Leiden, Netherlands; 2 National Institute of Parasitic Diseases, Chinese Center for Disease Control and Prevention, and Key Laboratory of Parasitology and Vector Biology, Ministry of Health, Shanghai, China; 3 Glycotechnology Laboratory, CICbiomaGUNE, San Sebastian, Spain; 4 CIBER-BBN, San Sebastian, Spain; 5 Centre for Immunology & Infection, Department of Biology, University of York, York, United Kingdom; Queen's University Belfast, IRELAND

## Abstract

**Background:**

Human immunity to *Schistosoma* infection requires many years of exposure, and multiple infections and treatments to develop. Unlike humans, rhesus macaques clear an established schistosome infection naturally at the same time acquiring immunity towards re-infection. In macaques, schistosome egg production decreases after 8 weeks post-infection and by week 22, physiological impairment of the worm caused by unclarified antibody-mediated processes is observed. Since strong antibody responses have been observed against schistosome glycan antigens in human and animal infections, we here investigate if anti-glycan antibodies are associated with immunity against schistosome infections in macaques.

**Methods:**

We used a microarray containing a large repertoire of glycoprotein- and glycolipid-derived glycans from different schistosome life stages to analyse anti-glycan serum IgG and IgM from *S*. *japonicum*-infected macaques during the course of infection and self-cure. We also used an *in vitro* schistosomula assay to investigate whether macaque sera containing anti-glycan antibodies can kill schistosomula.

**Conclusions/significance:**

Antibody responses towards schistosome glycans at week 4 post-infection were dominated by IgM while IgG was high at week 8. The profound increase in IgG was observed mainly for antibodies towards a large subset of glycans that contain (multi-)fucosylated terminal GalNAcβ1-4GlcNAc (LDN), and Galβ1-4(Fucα1–3)GlcNAc (LeX) motifs. In general, glycans with a higher degree of fucosylation gave rise to stronger antibody responses than non-fucosylated glycans. Interestingly, even though many IgG and IgM responses had declined by week 22 post-infection, IgG towards O-glycans with highly fucosylated LDN motifs remained. When incubating macaque serum with schistosomula *in vitro*, schistosomula death was positively correlated with the duration of infection of macaques; macaque serum taken 22 weeks post-infection caused most schistosomula to die, suggesting the presence of potentially protective antibodies. We hypothesize that IgGs against highly fucosylated LDN motifs that remain when the worms deteriorate are associated with infection clearance and the resistance to re-infection in macaques.

## Author summary

Schistosomes express many glycan antigens to which antibodies are raised by the infected host. These glycans may therefore form potential vaccine targets. Unlike humans where the disease persists chronically if not treated, schistosome-infected rhesus macaques are able to elicit a self-cure process naturally. To find out if anti-glycan responses could contribute to the natural clearance process, we followed the dynamics of anti-glycan serum antibodies in *Schistosoma*-infected macaques in a longitudinal study starting from the onset of infection until 22 weeks post-infection, when the macaques had eliminated most of the parasites. We found that sera of macaques taken after 22 weeks of infection contained high IgG titres towards specific schistosome glycan epitopes highly abundant on schistosome larvae. Moreover, infected macaque serum at week 22 was able to kill schistosomula *in vitro*. Our results suggest that anti-glycan antibodies play an important role in the self-cure process and the acquired resistance to re-infection in *Schistosoma* infected macaques.

## Introduction

Schistosomiasis is a debilitating parasitic disease caused by members of the helminth genus *Schistosoma (S*.*)*, with *S*. *mansoni*, *S*. *japonicum* and *S*. *haematobium* being the most prevalent human species. Once *Schistosoma* infection establishes, mature worms can live up to 30 years in the host until treated [1]. Many studies on human *Schistosoma* infection have indicated that resistance to *Schistosoma* infection can be acquired, but this is age-dependent and requires many years of exposure to the parasite, and multiple infections and treatments to develop [2]. Praziquantel (PZQ) is widely used to treat human schistosomiasis by paralyzing adult worm muscles and damaging the tegument [3]. This exposes worm antigens to the host immune system [4] and leads to immune-mediated killing of the parasite. The immune responses triggered by degenerating worms can alter antibody and cytokine responses and provide short-term drug-induced resistance to re-infection [5, 6]. Since this resistance is short-lived, people in endemic areas still require repeated administration of PZQ [7]. An effective elimination strategy would likely require the incorporation of a vaccine to immunize against schistosome (re)infection [8, 9].

Rhesus macaques are permissive hosts for *Schistosoma* infections. In rhesus macaques infected with *S*. *japonicum*, oviposition occurs 34–36 days after exposure and the peak egg excretion occurs 7–15 weeks post-infection [10]. However, unlike *Schistosoma* infections in humans where the infection persists with heavy egg shedding for decades, rhesus macaques show various signs of resistance to infection four months after infection [11]. Marked decrease in eggs detected in the faeces of macaques is observed 11 weeks post-infection, correlating to the vulnerable health status of the female worms, as seen by the diminished body lengths and size of sexual organs [10]. The rate of adult worm recovery from macaques also greatly decreases to 32% 19 weeks post-infection and 9% by the 42^nd^ week [10]. The same type of worm degeneration and diminished oviposition is observed in *S*. *mansoni*-infected rhesus macaques [12]. However, the decline in faecal egg output was observed at a slightly earlier time point: 9 weeks post-infection. In addition to eliminating the worms from the body, rhesus macaques were found to be completely resistant to secondary infection 21 weeks after primary infection when challenged with schistosome cercariae, even though the macaques were still in the process of clearing the primary infection [13]. It has been postulated that rhesus macaques clear schistosome infection and become resistant to re-infection through an antibody-mediated process, based on a strong inverse relationship observed between the intensity of IgG response and worm burden [12]. Additionally, when blood feeding worms were cultured *in vitro* with serum of macaques with low worm burden, stunted growth was observed for these worms. Moreover, it has been shown that serum antibodies from infected individuals can kill schistosomula *in vitro* [14, 15]. Recently, Li et al., have suggested that antibody binding to adult worm oesophagus blocks nutrient uptake and eventually lead to starvation of worms [16]. In view of the long time taken for the worms to degenerate, it is likely that the mechanism of clearance does not involve complement fixation [17] but a sustained antibody-mediated process that affects the normal physiology of worms.

An abundance of antibodies is generated in *Schistosoma*-infected hosts that bind to glycans from schistosome glycoproteins and glycolipids [18–24]. Localization studies with glycan-directed monoclonal antibodies [25, 26] and glycomic profiling by mass spectrometry [27] have indicated that *Schistosoma mansoni* glycosylation exhibits stage-specific changes during the life cycle. For example, the structural motifs Fucα1-3GalNAcβ1-4GlcNAc (F-LDN) and Fucα1-3GalNAcβ1-4(Fucα1–3)GlcNAc (F-LDN-F) are abundantly expressed in cercarial and egg glycoproteins but could hardly be detected in adult worm glycoproteins [26]. Nevertheless, multi-fucosylated GalNAcβ1-4GlcNAc (LDN) motifs are present in glycolipids throughout the whole life cycle. Cercarial N-glycans are found to be dominated by the Galβ1-4(Fucα1–3)GlcNAc (LeX) termini [28]. However, the expression of LeX by cercariae is rapidly lost after their transformation into schistosomula, while LDN motifs gradually become predominant in maturing worms [27]. While some glycan types and motifs are expressed in a stage-specific manner, cross-reactive glycans exist between different life stages. It has been shown that many antibodies elicited by egg glycans are cross-reactive with glycans expressed on the surface and secretions of cercariae [29]. Apart from cross-life stage similarities, there are also high cross-species similarities in schistosomes. *S*. *japonicum* and *S*. *mansoni* have a 86% homology in protein coding gene sequences [30] and *S*. *mansoni-*infected human sera recognize *S*. *japonicum* proteins on a schistosome protein array [31] and vice versa [32]. Although *S*. *mansoni* glycosylation is better characterized than that of *S*. *japonicum*, studies have suggested that the glycans expressed by the two species are highly similar [33, 34].

Recently, anti-glycan responses in *Schistosoma* infected humans and animals have been studied using glycan microarray approaches [19, 22–24]. With the aim of identifying possible glycan targets involved in clearance of *Schistosoma* and resistance to reinfection, we here analysed anti-glycan antibodies in a set of *S*. *japonicum-*infected rhesus macaque sera collected over a period of 22 weeks, using a microarray containing a large repertoire of N-, O- and glycosphingolipid (GSL) derived glycans isolated from *S*. *mansoni* larvae, adult worms and eggs [19, 23, 35] complemented with a synthetic microarray containing a set of relevant core-modified N-glycans [35]. After obtaining the antibody profile of schistosome-infected rhesus macaques at different infection stages, we incubated these sera with *in vitro* transformed schistosomula to study the effect of antibodies on parasite survival. Our work provides new insights into glycan motifs that may be the targets of a protective antibody response to *Schistosoma* infection.

## Materials and methods

### Ethics statement

The housing conditions, experimental procedures and animal welfare of the monkeys used in the study were in strict accordance with the national guidelines for the Care and Use of Animals established by the Chinese National Animal Research Authority and applied by the Institutional Animal Care and Use Committee (IACUC) of the Kunming Institute of Zoology, Chinese Academy of Sciences (CAS). The experimental protocol was approved by the Ethics Committee of Kunming Institute of Zoology, CAS (ID SYDW-2011017). The study used six adult male rhesus macaques (Macaca mulatta) from the captive-breeding colony at the Kunming Primate Research Center, CAS. They were group-housed prior to the experiment but then singly after infection for faecal sampling purposes. The separate cages were arranged in one large room to allow the monkeys visual, olfactory and auditory interactions with each other. Food and water were available ad libitum and vitamins were provided. The animals were also provided with environmental enrichment, such as toys designed especially for monkeys, to promote psychological well-being. The design and execution of the study complied with the recommendations of the Weatherall report (2006) on “The use of non-human primates in research”, which specifically mentions the continuing requirement for their use in schistosome research.

### Sera

Rhesus macaques were anaesthetised with ketamine hydrochloride (6 mg/kg body weight. Gutian Pharmaceutical Corporation, Fujian China) and infected percutaneously with 600 cercariae of *S*. *japonicum* via the shaved abdominal skin for 30 minutes. *S*. *japonicum* cercariae were obtained from patent *Oncomelania hupensis* snails kept at the Jiangsu Institute of Parasitic Diseases (Wuxi, China) [16]. Blood was obtained by intravenous sampling at 5 time points, week 0, 4, 8, 14 and 22, stood at room temperature for 1 hour (h) to clot, and kept overnight at 4°C to facilitate clot retraction before serum was recovered for storage at -20°C. All animals were individually inspected daily. Those showing signs of diarrhoea were given oral dehydration therapy as required

### Materials

Cy3 conjugated goat anti-human IgG (Fc-specific) and Alexa Fluor 647 conjugated goat anti-human IgM (μ chain specific) were from Invitrogen (The Netherlands). BSA and ethanolamine were from Sigma (Zwijndrecht, the Netherlands).

### Glycan microarray construction and analysis

The shotgun glycan microarray was constructed as described previously [19, 23]. A selection of fractions was analysed in this study compared to those previously described and contained reverse phase HPLC fractions of glycans isolated from cercariae (82 N-glycan fractions, 114 O-glycan fractions and 21 glycolipid glycans), adult worm (83 N-glycan fractions, 39 O-glycan fractions) and egg (62 egg N-glycan fractions, 110 soluble egg antigen O-glycan fractions and 12 glycolipid glycans). Additionally, 24 blank spots with spot buffer were included for array background control. Each glycan fraction was immobilized on a glass slide in triplicate.

The synthetic array used in this study contained a collection of core-xylosylated and core-α3 and α-6 fucosylated N-glycans with various core extensions [35]. The synthetic N-glycans were immobilized to N-hydroxysuccinimide (NHS)-activated glass slides via a C5 amino linker. Unreacted NHS groups were quenched by blocking with 50 mM ethanolamine in 50 mM sodium borate buffer, pH 9.0 for 1 h. The slides were then washed with PBST, PBS and MilliQ water, dried and then stored at -20°C. The slides were defrosted upon use, followed by serum sample incubation, as described in the following section.

### Binding assay

The glycan-microarray binding assay followed the protocol as described by van Diepen et al. [19, 23]. Briefly, the microarray slide was blocked with 2% BSA, 50 mM ethanolamine in PBS. Serum samples were diluted 1:100 in PBS-0.01% Tween20 with 1% BSA. Cy3-labeled anti-human IgG and Alexa Fluor 647-labeled anti-human IgM were diluted 1:1000 in PBS-0.01 Tween20 to detect bound serum antibodies on the slide. All washing steps were performed with successive rinses with PBS-0.05% Tween20 and with PBS. The last washing step was finished by an additional wash with milliQ water and the slides were dried and kept in the dark until scanning.

### Scanning and data analysis

A G2565BA scanner (Agilent Technologies, Santa Clara, CA) was used to scan the slides for fluorescence at 10 um resolution using lasers at 532 nm and 633 nm. Total IgG was detected at 532 nm and IgM at 633 nm, the 2-AA label does not fluoresce at these wavelengths. Data and image analysis was performed with GenePix Pro 7.0 software (Molecular Devices, Sunnyvale, CA). Spots were aligned and re-sized using round features with no CPI threshold. Background-subtracted median intensities were averaged per time point and processed as described by Oyelaran et al. [36]. Datasets were log_2_ transformed to remove the basic trends of variance. A hierarchical clustering analysis (HCA, complete linkage clustering using Euclidean distance metric) was performed to group associated glycan fractions using MultiExperiment Viewer v4.5. To identify statistically different IgG and IgM response towards glycan fractions, a paired sample t-test was performed using SPSS. A P value < 0.05 was used to identify glycan fractions that were differentially recognized by serum IgG and IgM antibodies.

### Schistosomula transformation

Freshly shed cercariae from snails were centrifuged at 440 × g for 5 min. The buffer was replaced with 37°C M199 medium supplemented with 1:100 1M HEPES pH7.4, 1x antibiotic antimycotic solution (ABAM), 1.5 mM glutamine and 10% FCS. The cercariae were resuspended with the medium and incubated at 37°C for 20 minutes in order to facilitate cercarial transformation into schistosomula. The incubated tube was shaken regularly to avoid sedimentation. Afterwards, the parasites were transferred to a petridish and put on an orbital shaker. During orbital shaking, schistosomula that collect at the centre of the petridish were taken out with a pipette, while swimming cercaria and tails that collect at the sides of the petridish were left behind. Isolated schistosomula were resuspended in supplemented M199 medium and cultured in a microtitre plate at 37°C in a humidified atmosphere with 5% CO_2_.

### Schistosomula killing assay

A total of 400 transformed schistosomula was cultured in each well of a flat bottom 96 well plate containing 100 ul of M199 medium supplemented with HEPES pH7.4, 1x antibiotic ABAM, 1.5mM glutamine and 10% FCS at 37°C in a humidified atmosphere with 5% CO_2_. At 3 hours post transformation, 55 ul of medium was carefully taken out of the wells and 5 ul of sera was added to each well to create a 1:10 serum dilution. Where required, sera were treated by incubation at 56°C for 45 minutes to inactivate complement. Each treatment was done in duplicate. Immediately after treatment, the plate was observed under a microscope to detect gross changes, such as schistosomula agglutination. The effect of treatment and induction of schistosomula killing was measured at 24 h and 48 h after treatment. Morphological changes were observed by using brightfield microscopy while schistosomula integrity was determined by fluorescent microscopy (Leica) using propidium iodide (PI) [37] staining at 10 ug/ml. Multiple photographs were taken of each well and the percentage of PI positive schistosomula was counted afterwards.

## Results

### IgG and IgM responses against schistosome glycans

**Fig 1:**
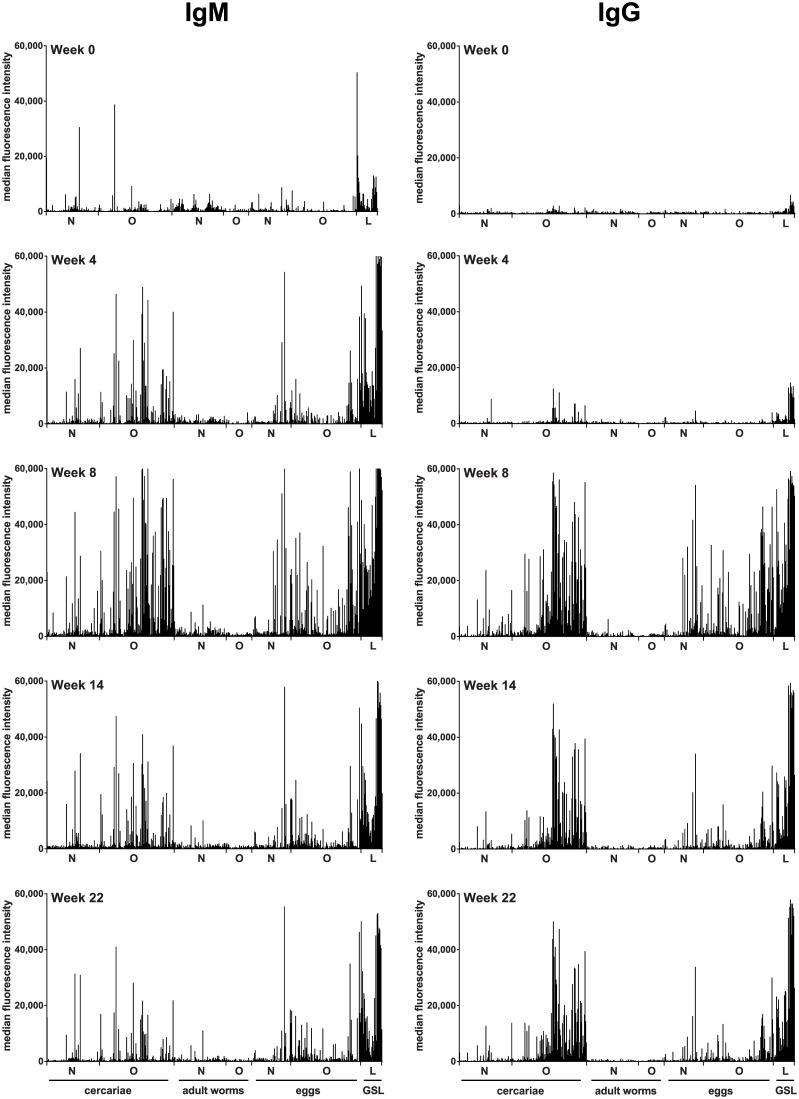
Averaged serum IgG and IgM response from schistosome-infected macaques to glycans isolated from different life stages of schistosomes. The horizontal axis indicates N- and O-glycan fractions from *Schistosoma* cercariae, adult worms, and eggs. Schistosome GSL glycans are shown as a group irrespective of the life stage. Average background-subtracted median fluorescence intensities are shown for IgG and IgM over a time course of 22 weeks. Each bar corresponds to antibody binding to individual glycan fractions printed on the glycan microarray. N: N-glycans. O: O-glycans and L: GSL glycans.

We incubated sera from six *S*. *japonicum*-infected rhesus macaques with a schistosome glycan microarray to investigate their anti-glycan antibody responses over a time course of 22 weeks. Fig 1 shows averaged IgG and IgM responses of rhesus macaques towards N-, O- and lipid-glycans isolated from *S*. *mansoni* cercariae, worms and eggs.

At the onset of infection, weak IgM signals against some glycans present on the schistosome array were detected in macaque serum, probably due to low levels of naturally occurring anti-glycan antibodies [38]. At week 4, strongly increased IgM was found binding to cercarial N- and O-glycans, egg-derived N-glycans and GSL glycans. Noteworthy, even though no eggs are produced 4 weeks post-infection, IgM antibodies were found against egg-derived glycans containing LeX and (fucosylated) LDN motifs; these glycan motifs have previously been shown to be shared with cercariae [26, 33, 34]. At 8 weeks post-infection, when oviposition was highest, maximum anti-glycan IgM titres were observed, especially against GSL-glycans and egg and cercarial O-glycans. These responses decreased at 14 weeks post-infection. At week 22, responses that persisted were very similar to those at week 4, but at a lower intensity. Notably, the IgM response at week 22 post-infection remained positive against a broad range of GSL-glycans.

In contrast to IgM, IgG of infected macaques directed towards schistosome glycans was negligible at week 0, while a slight induction of an IgG response against cercarial O-glycans and GSL-glycans could be detected at week 4 post-infection. At 8 weeks post-infection, a strong rise of IgG towards egg and cercarial N- and O-glycans was observed. Additionally, IgG against GSL glycans and cercarial O-glycans remained strongly positive at week 14 and week 22, while responses to egg O-glycans were greatly reduced during this period. *Schistosoma* GSL glycans had high IgG and IgM binding at week 22.

### IgG and IgM response profiles of *S*. *japonicum*-infected macaques

**Fig 2:**
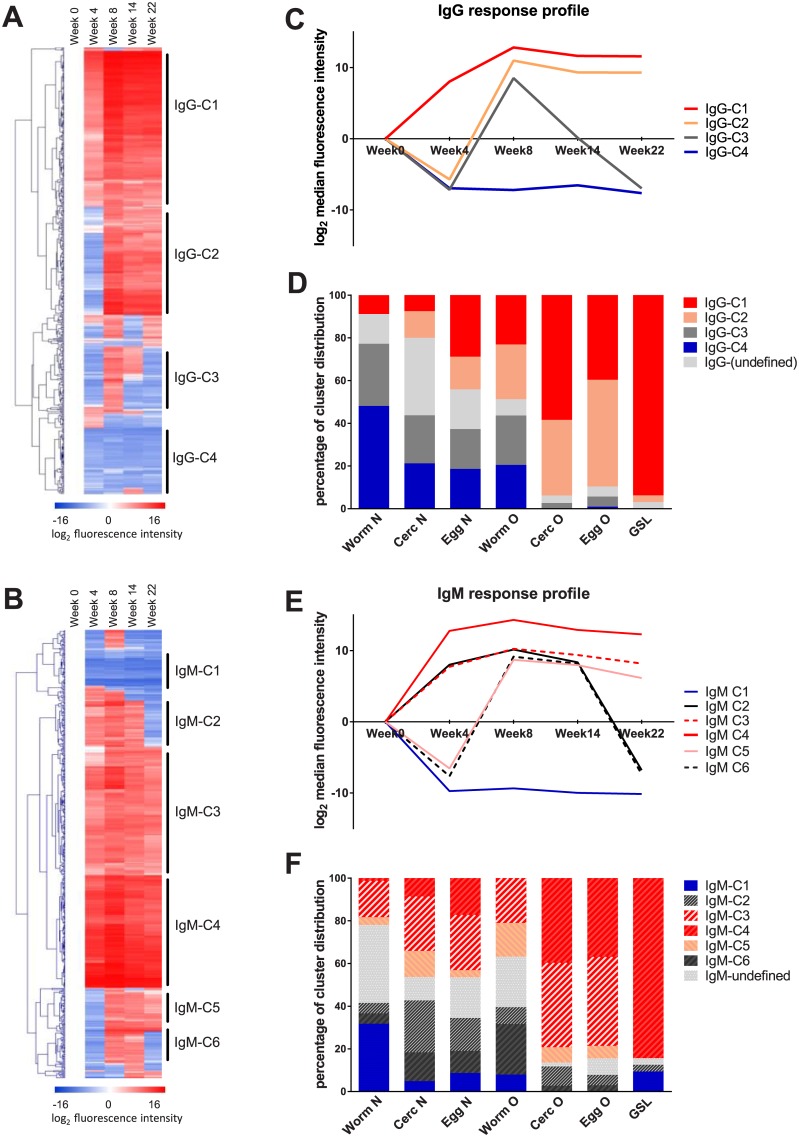
Hierarchical clustering analysis of macaque serum anti-glycan antibody responses over 22 weeks after infection with *Schistosoma japonicum*. Antibody median fluorescence intensity was corrected for baseline and log_2_ transformed. The reduction in antibody binding is indicated in blue, while the increase is indicated in red. Four major clusters were identified based on IgG response dynamics over time (A), and six major clusters for IgM (B). The median fluorescence intensity at each time point was averaged to obtain a response profile curve for each IgG (C) and IgM (E) major cluster. Distribution of IgG and IgM cluster profiles based on glycan origin is shown in (D) and (F), respectively. Worm N, worm derived N-glycan; Cerc N, cercaria derived N-glycan; Egg N, egg derived N-glycan; Worm O, worm derived O-glycan; Cerc O, cercaria derived O-glycan; Worm O, worm derived O-glycan; GSL, lipid derived glycan.

Following the IgG and IgM response patterns, we performed a hierarchical clustering analysis to group glycans with similar antibody response profiles. Anti-glycan antibody responses were corrected for baseline (week 0) intensity to obtain the intensity of response induced by infection. Four different glycan clusters were identified, based on IgG dynamics, namely IgG-C1, IgG-C2, IgG-C3 and IgG-C4 (Fig 2A) and six clusters for IgM dynamics, namely IgM-C1, IgM-C2, IgM-C3, IgM-C4, IgM-C5 and IgM-C6 (Fig 2B).

IgG-C1 contained glycans that were highly antigenic; antibodies against this cluster reached a maximum at week 8 post-infection, and remained until week 22 (Fig 2C). IgG-C2 elicited antibody responses that became positive between weeks 4 and 8, again remaining positive until week 22. In terms of antibody binding based on the detected fluorescence intensity of the array, there was a higher amount of IgG antibodies binding to glycans in IgG-C1 than in IgG-C2. Clusters IgG-C3 and IgG-C4 were smaller in size and glycans in these clusters did not yield high IgG signals. Antibodies against glycans in IgG-C3 peaked at week 8 post-infection and quickly decreased thereafter, while glycans in IgG-C4 did not appear to induce any IgG in macaques during the infection. A closer look at the type of glycans present in each cluster reveals that IgG-C1 and IgG-C2 were dominated by O-glycans and GSL-glycans, while IgG-C3 and IgG-C4 contained mostly N-glycans (Fig 2D and S1A Table).

Six response profiles were identified for IgM binding. There were three clusters that remained positive at week 22, namely IgM-C3, IgM-C4 and IgM-C5 (Fig 2E). Glycans in IgM-C3, -C4 and -C5 were mainly O-glycans derived from cercariae and eggs and lipid derived glycans (Fig 2F and S3A Table). On the other hand, clusters IgM-C1, -C2 and -C6, with no sustained antibody binding at week 22 post-infection, contained a majority of N-glycans. IgM-C1, -C2 and -C6 differed in the onset of IgM binding: while glycans in IgM-C2 were bound by macaque serum IgM generated at week 4 and later, glycans in IgM-C6 only had IgM binding at week 8. IgM-C1 had no infection-induced IgM binding at any timepoint.

Previously, the glycan composition of each glycan fraction printed on the array has been determined by mass spectrometry. Based on described structural glycan motifs in the literature [20, 23, 24, 27, 28, 33, 34, 39, 40], the most likely glycan structures for different glycan compositions were deduced for both IgG and IgM. We have summarized the most abundant glycan motifs for each IgG cluster, as well as the representative fractions in each IgG cluster in S1B and S2 Tables. The most common glycan motifs in IgG-C1 and IgG-C2 were the LeX and multi-fucosylated LDN motifs. O-glycan specific structural elements played an important role in the cluster formation of IgG-C4 and IgG-C2. For example, the *Schistosoma* specific O-glycan core Galβ1-3(Galβ1–6)GalNAc is sometimes found with an additional β1-6Gal on either the Galβ1–3 or the Galβ1–6 [41]. This structural element is found in cercariae-derived O-glycan fractions 3.4 and 6.6, and was one of the representative structures in IgG-C2 (S2 Table). Another example of an O-glycan specific motif was the multi-fucosylated Galβ1-4GalNAcβ1-4GlcNAc (Gal-LDN) motif [23, 42]. Compared to IgG-C2, IgG-C1 glycans were more complex and were in general longer in glycan chain length. For example, the di-LeX motif was expressed in both IgG-C1 and IgG-C2, but the tri-LeX motif was uniquely present in IgG-C1. In general, LDN motifs in IgG-C1 contained a higher extent of fucosylation; the previously defined DF-LDN-TF structure in cercarial O-glycan fraction 15.6 [23] was an antigenic motif uniquely found in cluster IgG-C1 (S2 Table).

In contrast to clusters IgG-C1 and IgG-C2 that were dominated by O–glycans (S1A Table), IgG-C3 and IgG-C4 consisted of 75% and 88% N-glycans, respectively, which were mostly worm-derived (Fig 2D). Most of the N-glycans in IgG-C4 did not have antigenic elements, but mainly expressed terminal Galβ1-4GlcNAc (LN), mannose and terminal GlcNAc (Gn) motifs (S2 Table). Compared to IgG-C4, IgG-C3 contained a higher number of fucosylated motifs, which led to a more antigenic response profile, although these responses were not sustained in time.

The clusters that had the highest amount of and long sustained IgM binding were IgM-C3 and -C4. The glycans in IgM-C3 and–C4 were abundant in antigenic motifs, such as LeX, (F)Gn, multiple fucosylated LDN and O-glycan-specific motifs β1–6 Gal and (fucosylated) Gal-LDN motifs (S3B Table). IgM-C5 was a small cluster that was also IgM positive at week 22, but had less IgM binding than IgM-C3 and IgM-C4. It contained 42% N-glycans, 58% O-glycans and no GSL glycans (S3A Table). IgM-C2 showed a response profile that was not recognized as a significant cluster in IgG. Glycans in IgM-C2 had IgM binding between week 4 and 14 and became undetectable at week 22; this cluster contained 64% N-glycans, 35% O-glycans and 2% lipid derived glycans with many glycans expressing the LeX motif.

When comparing IgG and IgM clusters, it was interesting to see that there was a sharp difference between the IgG antigenicity of N-glycans and O-glycans. A vast majority of N-glycans had no IgG binding at week 22 post-infection and were grouped in low antigenic clusters IgG-C3 and IgG-C4. On the other hand, IgM responses to many N-glycans were positive at week 22. A common pattern in IgG and IgM response profiles was that highly fucosylated glycan motifs led to higher serum antibody levels compared to less fucosylated structures. The more antigenic the cluster was, the more fucosylated motifs it contained. This effect was especially pronounced in IgG profiles.

### Infected macaque sera target core xylose/core α3 fucose

**Fig 3:**
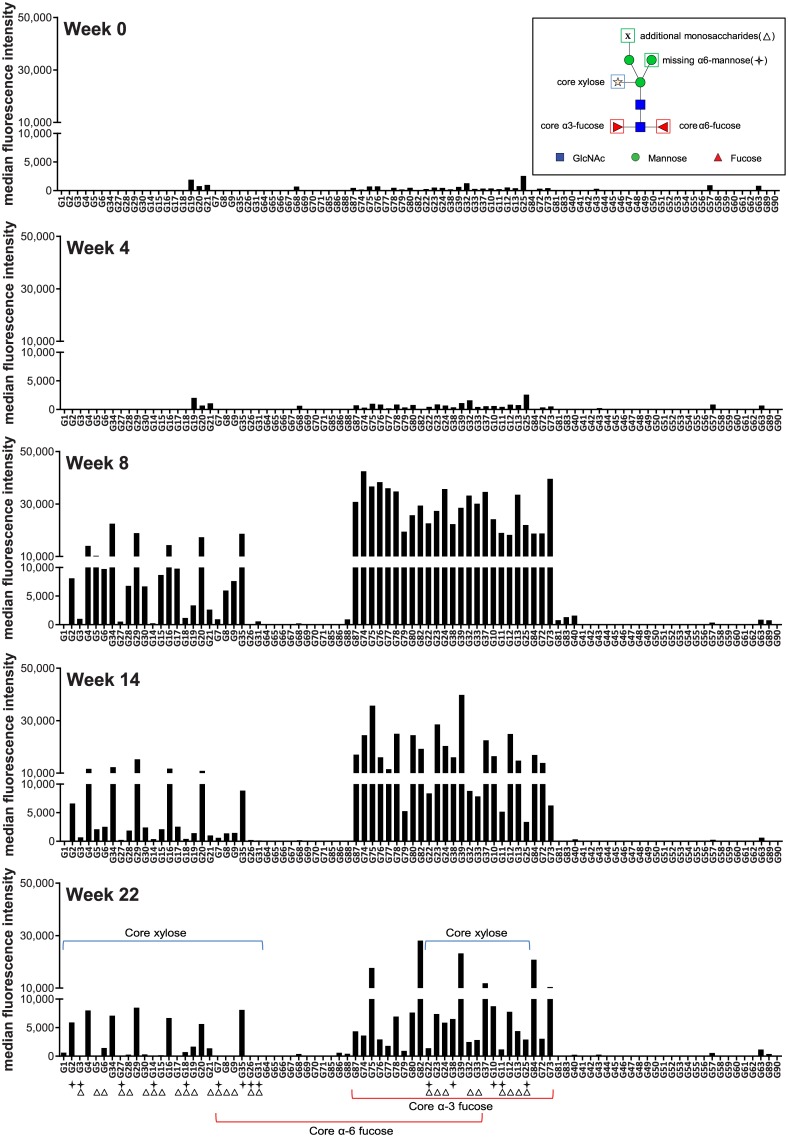
Averaged serum IgG responses from *S*. *japonicum*-infected rhesus macaques to synthetic core modified N-glycan fractions. The horizontal axis depicts core modified N-glycan fractions that have been synthesized and described by Brzezicka et al. [35]. Average median fluorescence intensities are shown for *S*. *japonicum*-infected macaque serum IgG over a time course of 22 weeks. Each peak along the vertical axis corresponds to each individual glycan fraction printed on the glycan microarray. Core xylosylated, core α6-fucosylated and core α3-fucosylated structures are indicated. Within core xylosylated structures, those that have additional monosaccharides on the α3-mannose (Δ) and those that miss the core α6-mannose (✧) are indicated.

In addition to the shotgun array, we tested the same set of macaque sera on a synthetic array previously described by Brzezicka et al.[35]. This array contains a collection of core-xylosylated and core-fucosylated N-glycans. Several of these defined synthetic glycan structures are present in schistosomes, including a number of core α3-fucose modified N-glycans which were not present on the shotgun array. Fig 3 shows IgG responses of macaque sera towards the synthetic glycan array over time (Fig 3). At week 0 and week 4 post-infection, there were minimal IgG responses against core α3-fucose and terminal LDN structures; the IgG response towards LDN on the α6 branch is stronger than towards LDN on the α3 branch. At week 8 post-infection, there was strongly increased IgG against structures with core xylose and core α3-fucose, either in combination with core α6-fucose, or alone. Interestingly, the absence of α6-mannose on xylosylated structures reduces IgG binding. Additionally, IgG binding was also reduced in xylosylated structures where the α3 branch was occupied by additional monosaccharides. At week 14 post-infection, a general decrease in IgG responses towards antigenic core modified glycans was observed. Those IgGs that remained at week 14 and 22 bound to unhindered core xylose and core α3-fucose-containing glycans. IgG binding to the xylosylated N-glycan core was similarly observed on the shotgun array peaking at week 8 post-infection and then decreasing (S1 Fig). Nevertheless, the amount of IgG binding to xylosylated structures was much lower than to other antigenic motifs on the same array, such as the fucosylated terminal LDN motifs. An interesting observation on the synthetic array was IgG binding to glycans G82 and G84, which had a Fucα1-3GlcNAcβ1,4(fucα1–3)GlcNAc modified N-glycan core that has been previously identified in *H*. *contortus* N-glycans [43] but not in schistosomes [27]. IgG towards G82 and G84 remained highly positive at week 22. We tested whether these glycans are recognized by an antibody against the structurally related Fucα1-3GalNAcβ1,4(fucα1–3)GlcNAc (F-LDN-F) motif, as the anti-F-LDN-F response is also sustained at week 22. However, anti-F-LDN-F monoclonal antibody 128-1E7 did not bind to G82 and G84 (S4 Fig), meaning that the Fucα1-3GlcNAcβ1,4 (Fucα1–3)GlcNAc modified N-glycan core is not cross-reactive with the F-LDN-F motif. It is likely that antibodies recognizing glycans G82 and G84 are recognizing core α3-fucose irrespective of the presence or absence of a second α3-fucose.

### Macaque sera at later infection time points kill schistosomula *in vitro* with higher effectiveness

**Fig 4:**
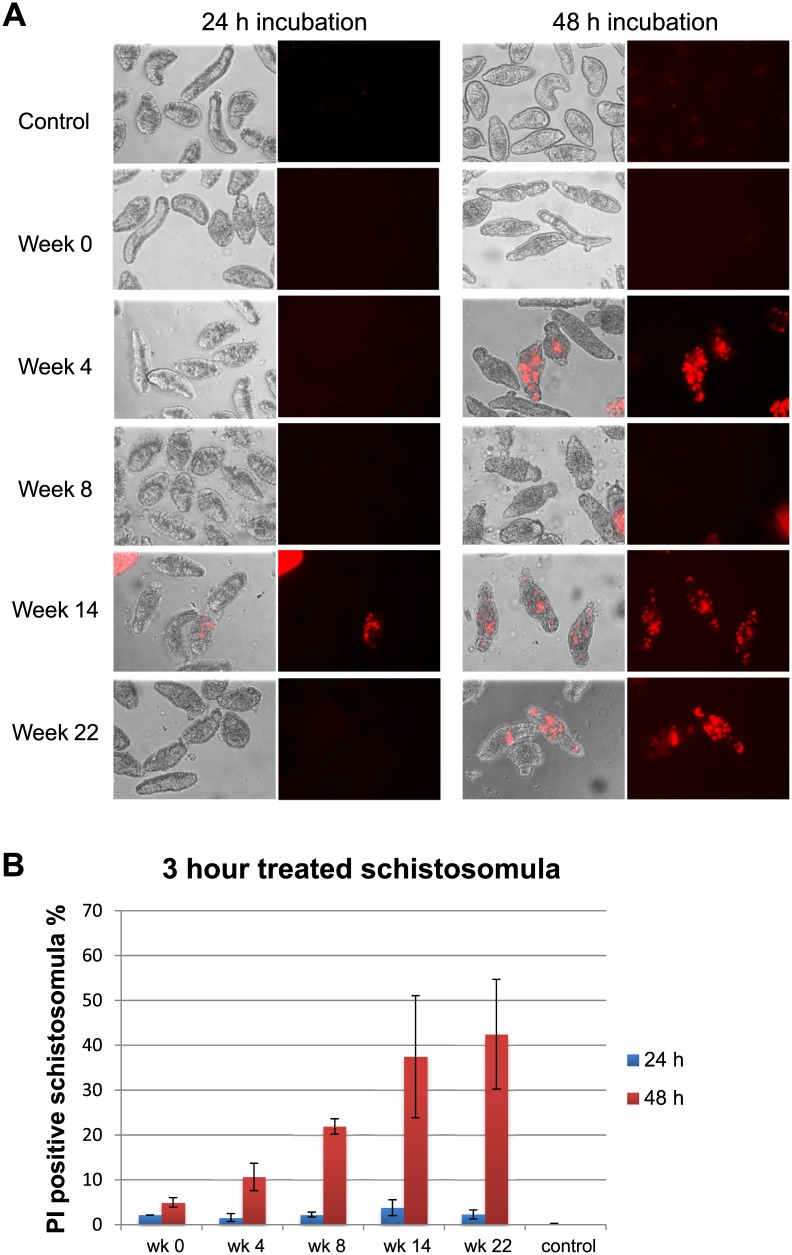
*In vitro* schistosomula incubation with *S*. *japonicum*-infected rhesus macaque sera collected at different infection time points. Macaque sera were incubated with 3 hour (h) transformed schistosomula. Loss of Schistosomula integrity was visualized by PI positivity. A) Gross morphology of schistosomula after 24 h and 48 h of incubation with macaque sera. B) Percentage of PI-positive schistosomula after 24 h and 48 h of incubation with macaque sera at different infection time points.

To investigate the potential functional involvement of macaque serum antibodies in resistance to infection by promoting schistosomula killing, we incubated sera collected at different infection time points with live *in vitro* transformed *S*. *mansoni* schistosomula. Schistosomula were treated 3 h post transformation with macaque sera (both heat inactivated and not heat inactivated) and their survival rates at 24 h and 48 h post treatment were determined. The schistosomula were visualized by brightfield microscopy and the viability was assessed by schistosomula integrity with propidium iodide (PI) staining [37]. Macaque infection sera at week 14 and week 22 caused patent agglutination of schistosomula quickly after contact, while week 0 and week 4 sera did not lead to any agglutination. Additionally, we observed a clear time point-dependent effect on gross morphology: schistosomula treated with infection sera at week 0 and week 4 resemble control schistosomula without serum addition after 24 h of incubation (Fig 4A). Incubation with sera taken at weeks 8, 14 and 22 caused irregularity of schistosomula surfaces after 24 h of incubation, which became even more pronounced after 48 h, with blebbing of the surface. Similarly, there was an infection time point-dependent relationship with PI positivity, where sera at later infection time points caused most schistosomula death as measured by PI positivity (Fig 4B). While 3 h transformed schistosomula treated with sera at week 0 resulted in 95% schistosomula survival, week 22 sera reduced survival to 57% after 48 h of incubation. Immunofluorescence microscopy has confirmed binding of macaque serum antibodies at late infection time points to the whole surface of schistosomula at 24 h and 48 h post *in vitro* transformation (S3 Fig). Complement factors did not play a role in schistosomula killing as heat-inactivated macaque sera did not lead to increased survival of schistosomula compared to untreated sera taken at the same time point, even though we still observed patent agglutination and blebbing of schistosomula treated with week 8, week 14 and week 22 heat-inactivated serum (S2 Fig).

### Glycans with IgG^high^ and IgM^low^ binding at week 22 have multiple fucosylated LDN motifs

**Fig 5:**
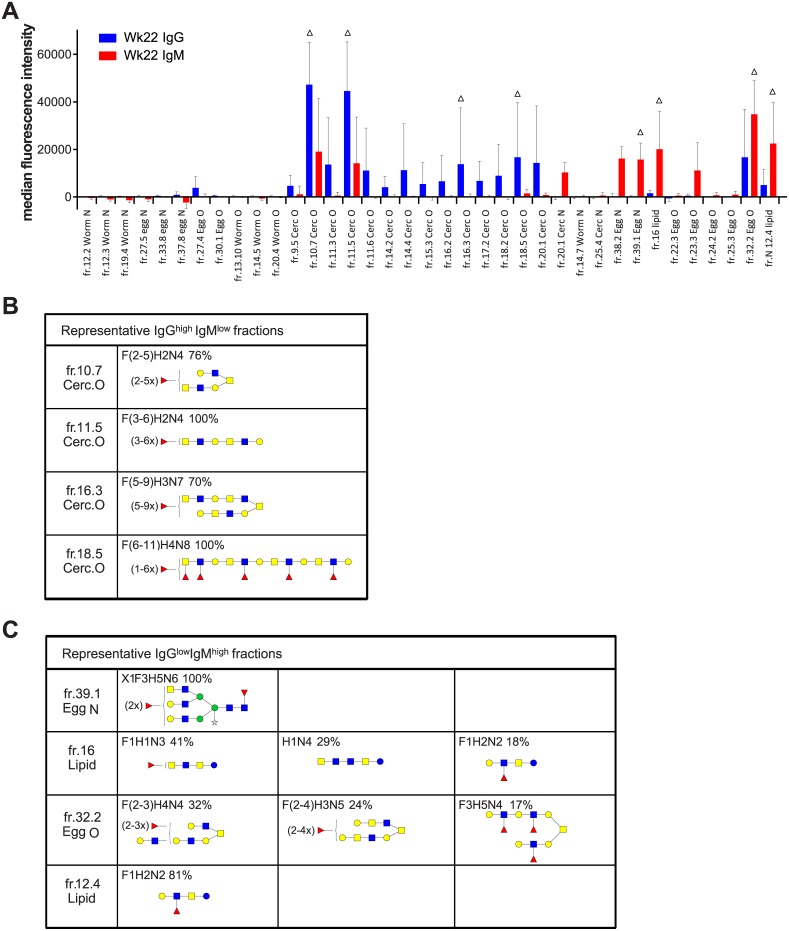
Glycan fractions that have statistically different serum IgG and IgM responses at week 22 post-infection. (A) 37 glycan fractions have a significant difference in IgG and IgM binding (p<0.05). Represented glycans are marked with Δ. (B) The glycan composition and most likely motif of representative fractions of IgG dominant and (C) IgM dominant groups are depicted.

Rhesus macaques are protected against a secondary schistosome infection 21 weeks after primary infection [13]. We observed that serum from macaques infected for 22 weeks had superior killing ability on 3 h *in vitro* transformed schistosomula compared to sera taken from earlier infection time points. The glycan array analyses showed that the IgG and IgM balance was changed at week 22 post-infection: while most anti-glycan IgMs have decreased, many anti-glycan IgG responses remained high. IgG is generally considered as the antibody isotype that provides effective protection to infection while IgMs are found to block the activity of protective IgGs by preventing effective antibody-dependent cell-mediated cytotoxicity (ADCC) of schistosomula *in vitro* [14]. We compared IgG and IgM response intensity at week 22 post-infection, to see which glycan fractions would have a difference in IgG and IgM responses, hypothesizing that glycan motifs which are IgG^high^ and IgM^low^ could be targets of protective immunity. We performed a statistical analysis on glycans that had infection-induced antibody binding at week 22 and found 37 glycan fractions that had a significant difference in IgG and IgM binding (Fig 5A). 12 of these fractions had higher IgM than IgG (IgG^low^IgM^high^), while 25 fractions had higher IgG than IgM (IgG^high^IgM^low^). We saw that the glycan fractions that were IgG^high^IgM^low^ almost all invariably contained a terminal multi-fucosylated LDN motif (Fig 5B). In contrast, glycans of the IgG^low^IgM^high^ group did not have highly fucosylated LDN structures and contained mostly LeX or LDN-F terminating motifs instead (Fig 5C). Five fractions were selected to represent each group (Fig 5B and 5C). Notably, 56% of the glycans in the IgG dominant group were cercarial O-glycans (S4 Table).

## Discussion

Using a microarray approach, we have followed the anti-glycan IgG and IgM responses in *S*. *japonicum*-infected rhesus macaques over a time course of 22 weeks, from the time of infection until the macaques have been reported to become resistant to reinfection while eliminating existing worms [13]. The schistosome array used in this study consisted of large repertoire of N-, O- and GSL derived glycans isolated from *S*. *mansoni* larvae, adult worms and eggs [19, 23]. A similar array has previously been used to study the different anti-glycan responses in adults and children in *S*. *mansoni* endemic regions [19, 23] and to study antibodies from local lymph node cells during *S*. *japonicum* infection of rats [24]. This array has been complemented with a previously established focussed synthetic array [35] in order to address the antigenic properties of a specific set of xylosyl and fucosyl N-glycan core modifications.

One question addressed in the current study was whether anti-glycan antibodies may be associated with the elimination of mature worms by *S*. *japonicum*-infected rhesus macaques. Previous studies have indicated that O-glycan expression in schistosome adult worms is limited in comparison to cercariae and eggs, and that antibodies against worm glycans are not very cross-reactive with the highly antigenic egg and cercarial glycans [23]. In line with this observation, we saw minimal antibodies against worm glycoprotein glycans. We have observed that the extent of glycan fucosylation is an important factor for triggering the host immune response. Unlike cercariae and eggs that express many different antigenic highly fucosylated glycans on both glycoprotein and glycolipids, adult worms express antigenic fucosylated glycans such as F-LDN and F-LDN-F predominantly on glycolipids [26, 27] and the less antigenic LDN-F and LeX motifs on specific worm N- and O-glycan subsets [44, 45]. The glycolipid associated F-LDN and F-LDN-F antigen can be found in undefined parenchymal spots or ducts inside the adult worm [26], though the exact function of these parenchymal spots and ducts is not understood. Notably, S*chistosoma* worms isolated from 22 week-infected rhesus macaques have diminished body lengths and reproductive organs [16]. It has previously been suggested that schistosome worm elimination occurs by macaque IgG attacking gut digestive enzymes, tegument surface hydrolases and antioxidant enzymes, eventually leading to worm death through cessation of blood feeding [12]. Recently, Li et al. have shown that rhesus IgG binds to the esophageal lumen of *S*. *japonicum* worms and co-localizes with esophageal secreted proteins, MEGs 4.1, 8.2, 9, 11 and VAL-7 [16]. It was suggested that rhesus IgGs block esophageal function making blood difficult to ingest, which eventually leads to the starvation of schistosome worms. It is not known whether glycans are the targets of these IgGs. Interestingly, esophageal located protein MEG-4.1 from *S*. *mansoni* was found to be heavily O-glycosylated [46] and MEGs 4.1 and 8.2 of *S*. *japonicum* are predicted to have similar properties [16]. It is noteworthy that three transcripts of glycosyltransferases were highly enriched in the male esophageal region but not in the posterior containing gut and tegument epithelia [47], which might indicate the synthesis of novel esophageal glycans. Unfortunately, esophageal located MEG proteins are expressed and secreted in minute amounts, and their O-glycans are not yet identified. If the esophageal MEG proteins carry unique O-glycans, it is unlikely that these are represented on the array due to their very low relative abundance in the overall worm glycome. Alternatively, MEGs may contain simple mono- and disaccharides such as GalNAc or Galβ1-3GalNAc (T and Tn antigens) that form multivalent O-glycosylated peptide domains. Monosaccharides and disaccharides are not isolated and printed using our glycan array methodology. If feasible, it would be more appropriate to determine binding of antibodies to short O-glycans structured in mucin domains in the context the native O-glycopeptide, or an identical synthetic construct thereof.

As well as eliminating adult worms, rhesus macaques are also found to be resistant towards secondary infection 16 weeks or more after primary exposure to schistosomes [13]. Freshly transformed schistosomula *in vitro* representing ‘skin stage’ schistosomula *in vivo* have previously been used to study mechanisms that might be related to reinfection [14, 15, 48]. Schistosomula are also susceptible to antibody-mediated damage [8, 49, 50] and may the best stage for the immune system to attack the parasite. Schistosomula-expressed glycans are a subset of glycans expressed by cercaria [27, 40]. We have shown that 3 h transformed schistosomula are killed by macaque sera in an infection time point-dependent manner: sera taken from macaques after 22 weeks of infection were more effective at killing than earlier infection time points, suggesting that the macaques may build up immunity towards the parasite during the infection. Luyai et al. have previously shown that rhesus sera from week 8 and week 11 post-infection were most efficient in killing schistosomula of *S*. *mansoni in vitro* [22]. However, we observed that macaque sera taken 14 and 22 weekspost-infection were even more effective in killing schistosomula compared to sera taken 8 weeks after infection.

At week 14 and week 22, the most prominent epitopes with high IgG binding were the highly fucosylated glycan motifs expressed on O-glycans and glycosphingolipid-derived glycans. Our statistical analysis on serum IgG and IgM balance at week 22 post-infection showed that glycans that were statistically IgG^high^IgM^low^ were mostly cercarial O-glycan fractions. Moreover, these IgG^high^IgM^low^ cercarial O-glycan fractions all contained highly fucosylated LDN epitopes. IgG is usually considered the protective antibody isotype compared to IgM. Early studies on human resistance to schistosome reinfection found a positive correlation between reinfection intensity and anti-schistosomula and anti-egg IgM antibodies [51, 52]. In addition, a strong inverse relationship is observed between the rapidity and intensity of IgG response and worm burden at 18 weeks in rhesus macaques [12]. We acknowledge that high IgG titres towards a certain glycan epitope do not necessarily lead to resistance to reinfection. Different IgG subtypes have been shown to vary in their potency in inducing eosinophil-mediated killing of schistosomula: human serum IgG1 and IgG3 have been found to be more potent isotypes to induce eosinophil-mediated cytotoxicity, whereas IgG4 antibodies are found to inhibit the cytotoxicity mediated by IgG1 and IgG3 [15]. IgG2 antibodies are only cytotoxic in the presence of activated eosinophils. Additionally, IgG2 and IgG4 were found to correlate with susceptibility to schistosome reinfection [52, 53]. Therefore, a protective response against skin schistosomula may not simply derive from high titres of a certain antibody isotype, but also likely from a balanced selective expression of protective antibodies and an absence of blocking antibodies. In our study, glycans in cluster IgG-C3 possessed IgG binding 8 weeks post-infection, but these IgG responses disappeared after 14 weeks post-infection when the macaques are immune to a secondary infection. The glycans in this profile were mostly N-glycans with motifs such as LN, LeX, LDN, α2-mannose, terminal Gn and terminal fucosylated Gn (S1B Table). We postulate that the disappearance of IgGs against these glycan motifs could be involved in the effective immune protection found in macaques. On the other hand, it is also intriguing to see that antibodies that are sustained after macaque immunity are also found to bind to these motifs. Taking into account the different properties of IgG subtypes, it would be relevant to investigate whether the IgG response against motifs that disappear during infection clearance are of a different subtype than the IgGs that are sustained when macaques are protected.

At week 22, our last serum collection time point, infected macaques are already resistant to secondary infection [10, 16]. We suggest that multi-fucosylated LDN motifs that are IgG^high^IgM^low^ may be involved in this resistance. However, Luyai et al. showed that macaque sera from week 78 post-infection could not kill freshly transformed schistosomula [22]. It would therefore be very interesting to test whether week 78 infected rhesus macaques lack the high antibody titres against highly fucosylated LDN epitopes. This would further indicate whether antibodies against highly fucosylated LDN epitopes are involved in resistance to reinfection in rhesus macaques or not. Our results using the synthetic glycan microarray are in accordance with Luyai et al.[22] that schistosome-infected macaques generate high IgG antibody responses to the core xylose and core α3-core fucose and LeX and LDN epitopes of N-glycans. Nevertheless, it is important to realise that core α3-core fucose has only been found in mature eggs but not in schistosomula of *S*. *mansoni* [27] or *S*. *japonicum* [28]. Therefore, we believe that antibodies against α3-core fucose do not contribute to schistosomula killing. Additionally, unlike the IgG response towards multi-fucosylted LDN motifs that is sustained with time, the IgG response towards α3-core fucose appears during oviposition and decreases when the adult worm stop producing eggs (Fig 3), further supporting that α3-core fucose is egg-derived.

Our study of *S*. *japonicum*-infected macaques was performed on a *S*. *mansoni*-derived glycan microarray. On the basis of high similarity in protein coding gene sequences between the two species [30] and cross species protein recognition by antibodies [31, 32], the glycosylation patterns of the two species are expected to be very similar. Accordingly, we observed extensive binding of *S*. *mansoni* derived glycans by serum antibodies from *S*. *japonicum-*infected rhesus macaques (Fig 1). Interestingly, although *S*. *japonicum* glycans were described as less fucosylated than *S*. *mansoni* [33, 34], our glycan array data shows that antibodies against multi-fucosylated glycans are elicited in *S*. *japonicum-*infected rhesus macaques, indicating that *S*. *japonicum* does produce such glycan antigens, including those than contain the Fucα1-2Fucα1- (DF) sequence.

Rhesus macaques generate a plethora of anti-glycan antibodies during *S*. *japonicum* infection. In this study, we hypothesized that glycan motifs that have high IgG binding could be associated with resistance to *Schistosoma* infection. It appears that the presence of IgG antibodies against multi-fucosylated LDN is correlated with the effectiveness of schistosomula killing *in vitro*. However, it is interesting to see that humans that are generally susceptible to re-infection also generate antibodies towards many of these fucosylated glycans, although to a lower extent [19]. Although this observation certainly does not undermine the possible protective effect of anti-glycan antibodies, it indicates that the presence of antibodies towards certain epitopes is not directly related to protection. As discussed earlier, different studies have shown that antibody isotype balance affects resistance to reinfection [15, 51, 52]. Perhaps even in an unprotected infected host, protective antibodies exist, but not in sufficient quantities to overcome the infection, or protective antibodies are overshadowed by the abundance of irrelevant or blocking antibodies. Some researchers have suggested that high antibody responses towards glycans are beneficial not for the host, but for the parasite, by directing the immune system away from epitopes that could provide protective immunity [18, 29]. Mice vaccinated with viable schistosome eggs although eliciting high anti-glycan antibody titres, were not protected against cercarial challenge *in vivo* [18]. In our case, at least, we have shown that high antibody titres against glycans did not prevent rhesus macaques from eliminating schistosome adult worms while gaining resistance to reinfection. It is imperative to consider that macaques may have intrinsic antibody differences, rather than epitope specificity, that lead to protection. Antibody responses towards schistosome glycans in *S*. *japonicum-*infected rhesus macaques are very dynamic and worth further detailed study. Deciphering the specificity of antibodies sustained at week 22 post-infection may provide clues to the composition of the antibody pool in a host resistant to schistosome re-infection and may provide valuable information about the glycan targets that could be involved in protection against re-infection.

## Supporting information

S1 Table Distribution of glycan origin and putative glycan motifs present in each IgG glycan cluster. (PDF)

S2 Table (PDF)

S3 Table Distribution of glycan origin and putative glycan motifs present in each IgM glycan cluster. (PDF)

S4 Table Glycan origins and putative glycan motifs present in the IgG^high^IgM^low^ and IgG^low^IgM^high^ group. (PDF)

S1 Fig IgG response profile of different glycan motifs. (PDF)

S2 Fig *In vitro* schistosomula incubation with heat inactivated *S*. *japonicum*-infected macaque sera. (PDF)

S3 Fig Binding of *S*. *japonicum*-infected macaque serum antibodies to schistosomula. (PDF)

S4 Fig Binding of monoclonal antibody 128-1E7-C to a collection of synthetic glycans. (PDF)
