# Supplementary material for: Specific anti-glycan antibodies are sustained during and after parasite clearance in Schistosoma japonicum-infected rhesus macaques
Source: PLoS Negl Trop Dis. 2017 Feb 2;11(2):e0005339. doi: 10.1371/journal.pntd.0005339 (PMC5308859; doi:10.1371/journal.pntd.0005339)
Supplement: S1 Table — (PDF) [file pntd.0005339.s001.pdf]

S1 Table

A

Distribution of glycan origin in each IgG glycan cluster

	IgG-C1		IgG-C2		IgG-C3		IgG-C4	
worm N	4%		0%		33%		51%	
cerc N	3%	17%	8%	15%	26%	75%	23%	88%
egg N	10%		7%		16%		15%	
worm O	5%		8%		13%		11%	
cerc O	37%	66%	33%	84%	4%	25%	0%	12%
egg O	24%		43%		7%		1%	
GSL		17%		1%		0%		0%

B

Putative glycan motifs present in each IgG glycan cluster

Putative epitopes		IgG-C1	IgG-C2	IgG-C3	IgG-C4
Size of cluster		177 fractions	123 fractions	69 fractions	75 fractions
Core α6-fucose	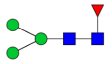	6%	7%	32%	41%
Xylose	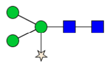	4%	7%	9%	11%
LN	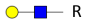	15%	24%	29%	27%
LeX	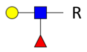	33%	34%	23%	13%
Di-LeX	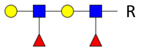	10%	7%	7%	1%
Tri-LeX	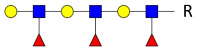	2%	0%	0%	0%
LDN	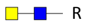	10%	12%	16%	17%
LDN(F=1)	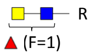	12%	14%	3%	0%
LDN(F≥2)	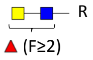	25%	10%	1%	0%
α2-Mannose	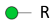	9%	7%	30%	36%
Gn	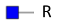	19%	31%	26%	20%
Gn(F≥1)	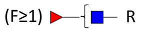	20%	30%	13%	4%
β1-6 gal	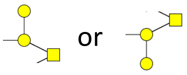	8%	12%	1%	0%
Gal-LDN	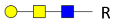	6%	12%	0%	0%
Gal-LDN (F≥1)	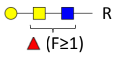	6%	13%	0%	0%

The most likely glycan motifs were depicted for each glycan fraction present in individual clusters. The percentages of each glycan motif present in each glycan cluster is shown. For most of the glycan fraction, more than one possible motif is present.
