# Supplementary material for: Specific anti-glycan antibodies are sustained during and after parasite clearance in Schistosoma japonicum-infected rhesus macaques
Source: PLoS Negl Trop Dis. 2017 Feb 2;11(2):e0005339. doi: 10.1371/journal.pntd.0005339 (PMC5308859; doi:10.1371/journal.pntd.0005339)
Supplement: S2 Table — (PDF) [file pntd.0005339.s002.pdf]

S2 Table

IgG-C1

Name of fraction[a]	Composition of abundant glycans in the fraction[b]	Putative glycan motifs[c]	Likely structure of the most abundant glycan in fraction[d]
*Cerc O fr.12.7	F5H2N5 35%	Gal-LDN(F0-5), LDN(F0-5)	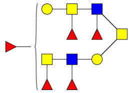
	F6H2N5 33%	Gal-LDN(F0-6), LDN(F0-6)	
	F8H2N5 23%	Gal-LDN(F2-6), LDN(F2-6)	
+Cerc O fr.15.6	F7H3N6 33%	DF-LDN-TF	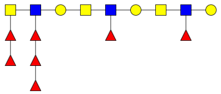
	F9H3N6 29%	DF-LDN-TF	
	F6H3N6 18%	DF-LDN-DF	
	F5H3N6 10%	DF-LDN-F	
Lipid L23.1	F4H1N6 73%	LDN(F0-4)	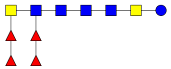
Lipid L24.1	F7H1N6 43%	LDN(F2-7)	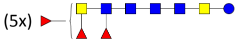
	F4H1N6 19%	LDN(0-4)	
	F8H1N6 15%	LDN(F2-8)	
*Egg O fr.32.2	F3H4N4 28%	LeX, Di-LeX, Tri-LeX, FGn	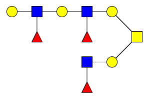 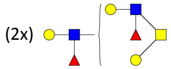 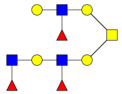
	F3H5N4 17%	LeX, Di-LeX, Tri-LeX	
	F3H3N5 16%	Gal-LDN(F0-3)	
*Egg O fr.36.2	F4H5N5 15%	LeX, Di-LeX, Tri-LeX, (F)Gn	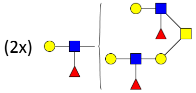 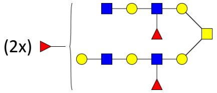
	F4H4N6 11%	FGn	

IgG-C2

Name of fraction[a]	Composition of abundant glycans in the fraction[b]	Putative glycan motifs[c]	Likely structure of the most abundant glycan in fraction[d]
Egg O fr.16.3	F2H1N3 100%	LDN(F2), (F0-2)Gn	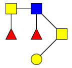 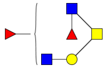
Egg O fr.27.2	F3H2N4 54%	(F0-2)Gn, Gal-LDN(F0-3)	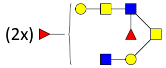
Cerc O fr.8.11	F4H1N3 86%	LDN(F4), (F1-3)Gn	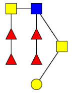 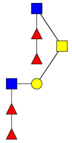
	F1H5N3 14%	β1-6 Gal, LeX, LN	
Cerc O fr.9.9	H3N5 42%	Gal-LDN	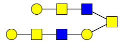
	H4N2 17%	LN, β1-6 Gal, Gn	
	F4H1N3 16%	LDN(F4), (F1-3)Gn	
	F1H4N2 14%	LeX, β1-6 Gal	
Cerc O fr.3.4	H4N2 100%	β1-6 Gal, LN, Gn	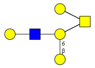 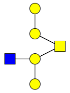
Cerc O fr.6.6	F1H3N1 100%	β1-6 Gal, LeX	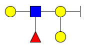
Egg N fr.38.2	X1F3H5N6 51%	Core α6-fucose, Xylose, LeX, LDN(F0-1), LN	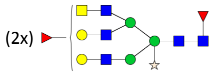
	X1F1H8N3 49%	Hybrid, Oligomannose, LN	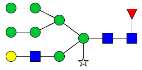

S2 Table

IgG-C3

Name of fraction[a]	Composition of abundant glycans in the fraction[b]	Putative glycan motifs[c]	Likely structure of the most abundant glycan in fraction[d]
Cerc N fr.25.4	F2H3N4 92%	Core α6-fucose, LDN(F1), (F)Gn	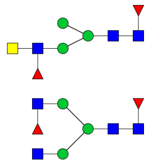
Egg O fr.26.2	F2H3N3 91%	LeX, diLeX, FGn	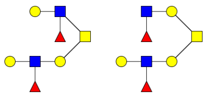
Worm N fr.15.6	F2H4N4 39%<br>F1H6N2 35%<br>F1H3N6 11%	Core α6-fucose, LN, LeX, (F)Gn<br>Core α6-fucose, oligo-mannose, LDN	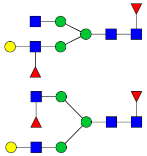
Cerc N fr.32.4	X1F3H5N4 100%	Core α6-fucose, LeX, Core xylose	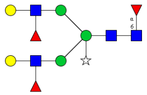
egg N fr.26.5	F1H4N4 53%<br>X1F1H3N5 47%	Core α6-fucose, Gn, LN<br>Core α6-fucose, LDN, Gn, xylose	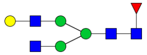
egg N fr.32.6	H3N7 67%<br>H6N5 33%	LDN, Gn<br>LN	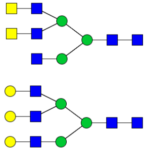
Worm N fr.19.7	H8N2 100%	Oligo-mannose	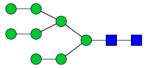

IgG-C4

Name of fraction[a]	Composition of abundant glycans in the fraction[b]	Putative glycan motifs[c]	Likely structure of the most abundant glycan in fraction[d]
Egg N fr.23.5	X1F1H3N4 39%<br>F1H4N4 17%<br>F1H4N3 16%	Core α6-fucose, LDN, Gn, xylose<br>Core α6-fucose, Gn, LN<br>Core α6-fucose, LN	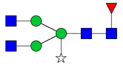
Worm N fr.16.7	F1H4N5 100%	Core α6-fucose, LN, LDN	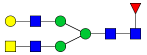
Cerc N fr.18.6	F1H3N3 100%	Core α6-fucose, Gn	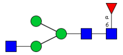
Worm N fr.10.2	H5N2 58%<br>H3N4 12%<br>F1H3N4 15%	Tri-mannosyl<br>LDN, Gn<br>Core α6-fucose, LDN, Gn	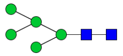
Worm N fr.18.6	H7N2 100%	olligomannose	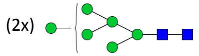
Cerc N fr.26.2	F2H4N3 100%	Core α6-fucose, LeX	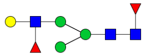

[a] Cerc N, cercarial derived N- glycan; Worm N, worm derived N- glycan; egg N-, egg derived N-glycan. Cerc O-, cercarial derived O- glycan; Worm O, worm derived O- glycan; lipid, lipid derived-glycan. Fraction numbers are also shown

[b] F, fucose; H, hexose; N, N-acetylhexosamine

[c] putative glycan motifs are proposed on the basis of composition of glycan fractions aided by literature (van Diepen, A., van der Plas, A.J., Kozak, R.P., Royle, L., Dunne, D.W., Hokke, C.H.,2015; Jang-Lee, J., Curwen, R. S., Ashton, P. D., et al., 2007)

[d]Most likely structure of the most abundant glycan in the fraction is shown. For the less abundant glycans in each fraction, only proposed motifs are depicted

*fractions previously described by (van Diepen, A et al., 2015) including monoclonal anti-glycan antibody results

+MALDI-TOF-MS/MS spectra of glycans detected in Schistosoma mansoni cercarial O- glycan fraction 15.6 shown in paper by van Diepen, A et al., 2015.
