# Supplementary material for: Specific anti-glycan antibodies are sustained during and after parasite clearance in Schistosoma japonicum-infected rhesus macaques
Source: PLoS Negl Trop Dis. 2017 Feb 2;11(2):e0005339. doi: 10.1371/journal.pntd.0005339 (PMC5308859; doi:10.1371/journal.pntd.0005339)
Supplement: S3 Table — (PDF) [file pntd.0005339.s003.pdf]

S3 Table

A

Distribution of glycan origin in each IgM glycan cluster

	IgM-C1		IgM-C2		IgM-C3		IgM-C4		IgM-C5		IgM-C6	
worm N	63%		8%		10%		1%		9%		11%	
cerc N	10%	85%	38%	63%	14%	34%	6%	14%	29%	43%	31%	58%
egg N	12%		17%		10%		8%		6%		17%	
worm O	7%		6%		6%	66%	0%		17%		25%	
cerc O	0%	7%	19%	35%	30%		35%	65%	23%	57%	8%	42%
egg O	0%		10%		30%		30%		17%		8%	
GSL		7%		2%		0%		21%		0%		0%

B

Putative glycan motifs present in each IgM glycan cluster

Putative epitopes		IgM-C1	IgM-C2	IgM-C3	IgM-C4	IgM-C5	IgM-C6
Size of cluster		41 fractions	52 fractions	145 fractions	127 fractions	35 fractions	36 fractions
Core α6-fucose	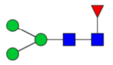	34%	29%	15%	9%	23%	17%
Xylose	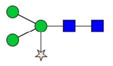	10%	17%	0%	7%	9%	11%
LN	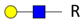	20%	29%	22%	16%	23%	31%
LeX	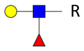	5%	29%	34%	28%	31%	22%
Di-LeX	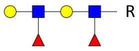	0%	12%	9%	6%	9%	6%
Tri-LeX	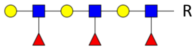	0%	2%	0%	2%	0%	0%
LDN	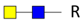	24%	8%	12%	9%	9%	11%
LDN(F=1)	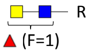	5%	4%	11%	15%	6%	3%
LDN(F≥2)	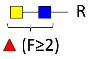	0%	6%	9%	29%	9%	0%
α2-Mannose	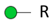	32%	27%	11%	4%	17%	25%
Gn	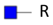	24%	17%	29%	14%	20%	25%
Gn(F≥1)	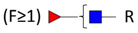	0%	8%	26%	19%	20%	14%
β1-6 gal	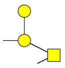 or 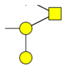	0%	6%	9%	8%	6%	6%
Gal-LDN	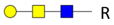	0%	4%	8%	9%	0%	3%
Gal-LDN(F≥1)	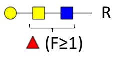	0%	2%	9%	10%	0%	3%

The most likely glycan motifs were depicted for each glycan fraction present in individual clusters. The percentages of each glycan motif present in each glycan cluster is shown. For most of the glycan fraction, more than one possible motif is present.
