# Supplementary material for: Specific anti-glycan antibodies are sustained during and after parasite clearance in Schistosoma japonicum-infected rhesus macaques
Source: PLoS Negl Trop Dis. 2017 Feb 2;11(2):e0005339. doi: 10.1371/journal.pntd.0005339 (PMC5308859; doi:10.1371/journal.pntd.0005339)
Supplement: S4 Table — (PDF) [file pntd.0005339.s004.pdf]

S4 Table

A

Glycan origins present in the IgG^{high}IgM^{low} and IgG^{low}IgM^{high} group

	IgG^{high}IgM^{low}		IgG^{low}IgM^{high}	
Size of cluster	25 fractions		12 fractions	
worm N	12%		8%	
cerc N	0%	24%	17%	42%
egg N	12%		17%	
worm O	12%		0%	
cerc O	56%	76%	0%	42%
egg O	8%		42%	
lipid		0%		17%

Fractions that had a significant IgG and IgM response difference were grouped into the IgG^{high}IgM^{low} (IgG response>IgM response) or the IgG^{low}IgM^{high} (IgM response>IgG response) group. The IgG^{high}IgM^{low} group was characterized by O- glycans, in particular cercariae derived O- glycans.

B

Putative glycan motifs present in the IgG^{high}IgM^{low} and IgG^{low}IgM^{high} group

Putative epitopes		IgG^{high}IgM^{low}	IgG^{low}IgM^{high}
Size of cluster		25 fractions	12 fractions
Core α6-fucose	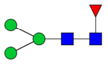	12%	42%
Xylose	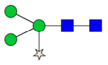	4%	17%
LN	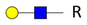	20%	17%
LeX	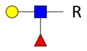	16%	33%
Di-LeX	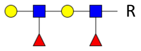	12%	17%
Tri-LeX	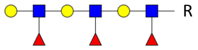	0%	8%
LDN	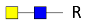	36%	25%
LDN(F=1)	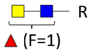	32%	33%
LDN(F≥2)	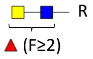	44%	0%
α2-Mannose	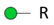	8%	0%
Gn	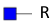	32%	42%
Gn(F≥1)	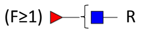	12%	50%
β1-6 gal	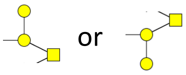	12%	0%
Gal-LDN	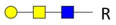	12%	25%
Gal-LDN(F≥1)	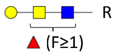	12%	25%

Glycan Motifs present in the IgG^{high}IgM^{low} and IgG^{low}IgM^{high} group.
