# Supplementary material for: Specific anti-glycan antibodies are sustained during and after parasite clearance in Schistosoma japonicum-infected rhesus macaques
Source: PLoS Negl Trop Dis. 2017 Feb 2;11(2):e0005339. doi: 10.1371/journal.pntd.0005339 (PMC5308859; doi:10.1371/journal.pntd.0005339)
Supplement: S1 Fig — (PDF) [file pntd.0005339.s005.pdf]

S1 Fig

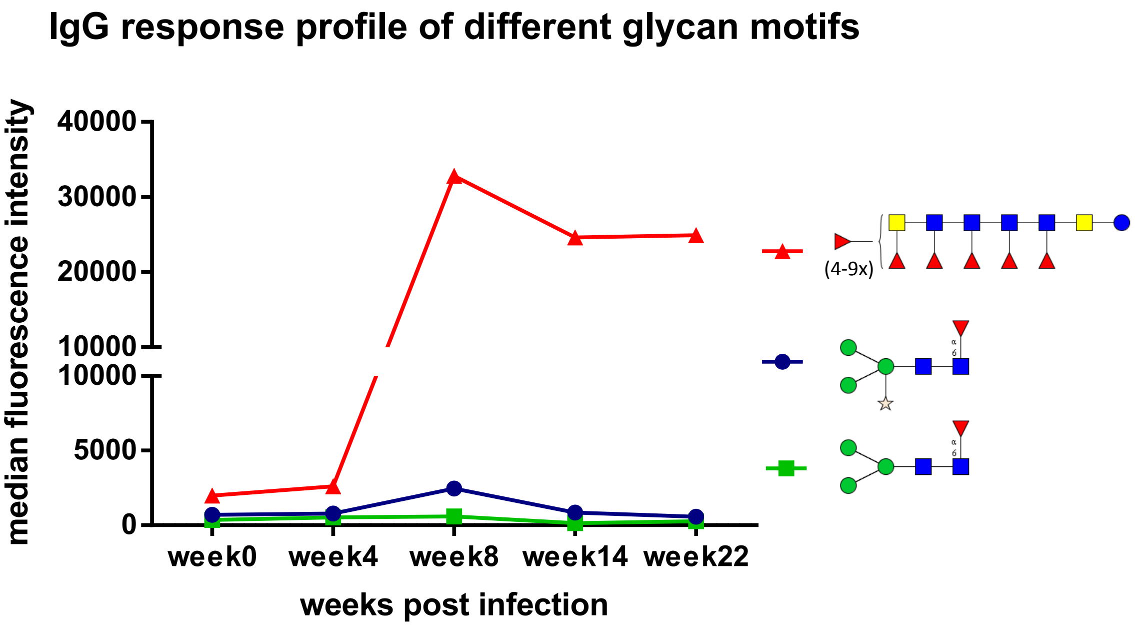

Average median fluorescence intensities are shown for *S. japonicum*-infected macaque serum IgG over a time course of 22 weeks towards different glycan motifs.
