# Supplementary material for: Specific anti-glycan antibodies are sustained during and after parasite clearance in Schistosoma japonicum-infected rhesus macaques
Source: PLoS Negl Trop Dis. 2017 Feb 2;11(2):e0005339. doi: 10.1371/journal.pntd.0005339 (PMC5308859; doi:10.1371/journal.pntd.0005339)
Supplement: S2 Fig — (PDF) [file pntd.0005339.s006.pdf]

S2 Fig

In vitro schistosomula incubation with heat inactivated _S. japonicum_-infected macaque sera

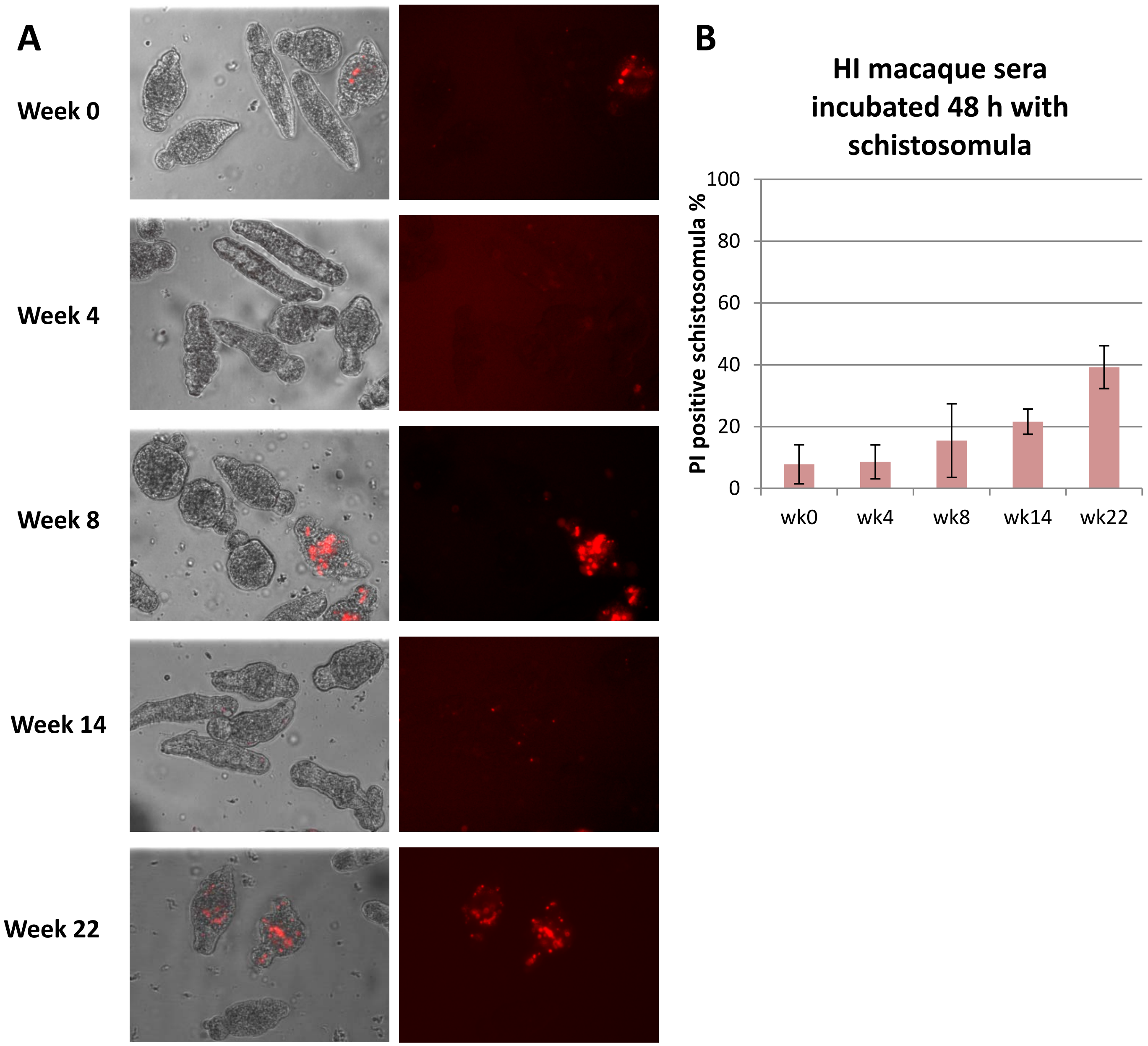

In vitro schistosomula incubation with heat inactivated (HI) *S. japonicum*-infected rhesus macaque sera collected at different infection time points.

HI Macaque sera were incubated with 3 h transformed schistosomula. Loss of Schistosomula integrity was visualized by PI positivity. A) Gross morphology of schistosomula after 48 h of incubation with HI macaque sera. B) Percentage of PI-positive schistosomula after 48 h of incubation with HI macaque sera at different infection time points.
