# Supplementary material for: Specific anti-glycan antibodies are sustained during and after parasite clearance in Schistosoma japonicum-infected rhesus macaques
Source: PLoS Negl Trop Dis. 2017 Feb 2;11(2):e0005339. doi: 10.1371/journal.pntd.0005339 (PMC5308859; doi:10.1371/journal.pntd.0005339)
Supplement: S3 Fig — (PDF) [file pntd.0005339.s007.pdf]

S3 Fig

Binding of *S. japonicum*-infected macaque serum antibodies to schistosomula

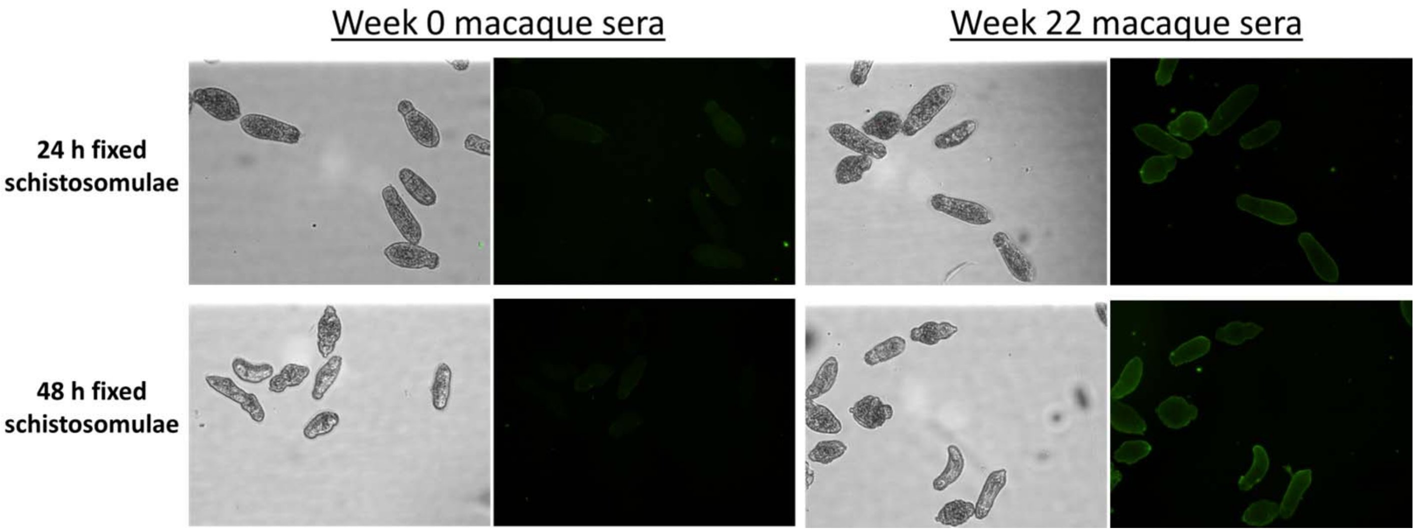

Schistosomula were fixed 24 h and 48 h after transformation in 2% PFA. Fixed schistosomula were washed twice in 200 μl PBS and then incubated with 0.5 μl of macaque serum (before infection and after 22 weeks of infection) in a suspension of 30 μl for 30 minutes. Parasites were washed 1 time with PBS and then suspended in 25 μl of 50x diluted rabbit-anti-human IgG, IgM IgA antibodies (DAKOPATTS) for 30 minutes. Afterwards, parasites were washed 3 times with PBS and suspended in 25 μl of 200x diluted Alexa fluor 488-conjugated goat-anti-rabbit IgG (H+L) antibody (invitrogen) for 30 minutes. Finally, the parasites were washed once in PBS and analyzed by fluorescence microscopy.
