# Supplementary material for: Specific anti-glycan antibodies are sustained during and after parasite clearance in Schistosoma japonicum-infected rhesus macaques
Source: PLoS Negl Trop Dis. 2017 Feb 2;11(2):e0005339. doi: 10.1371/journal.pntd.0005339 (PMC5308859; doi:10.1371/journal.pntd.0005339)
Supplement: S4 Fig — (PDF) [file pntd.0005339.s008.pdf]

S4 Fig

Binding of monoclonal antibody 128-1E7-C to a collection of synthetic glycans

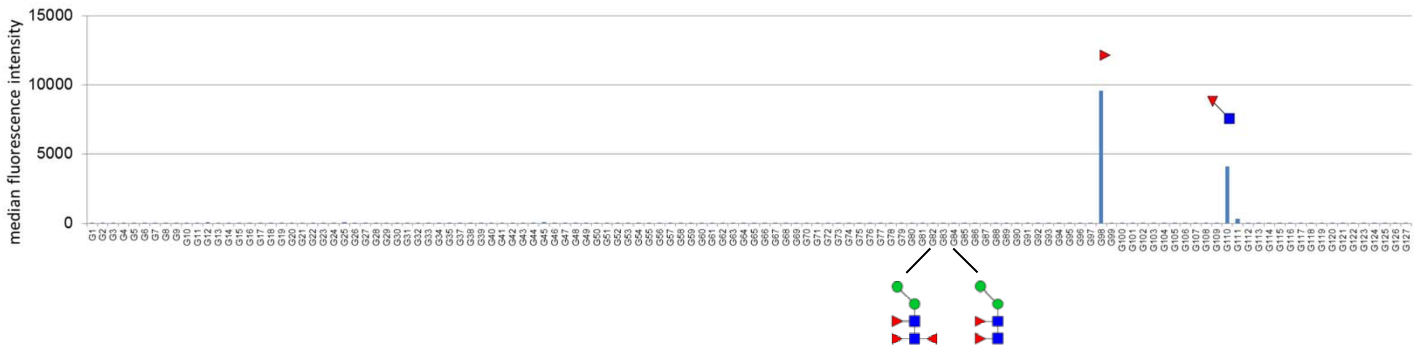

128-1E7-C is a monoclonal antibody that binds to the Fucα1-3GalNAcβ1,4(fucα1-3)GlcNAc (F-LDN-F) motif (Smit, C. H., A. Homann, V. P. van Hensbergen, G. Schramm, H. Haas, A. van Diepen and C. H. Hokke, 2015). In this figure, the horizontal axis depicts a collection of other glycans that have previously been synthesized and described by us (Brzezicka, K., B. Echeverria, S. Serna, A. van Diepen, C. H. Hokke and N.-C. Reichardt, 2015). The same binding assay protocol described in the Materials and Methods section was used to detect binding of 128-1E7-C to the glycan microarray. 128-1E7-C was incubated at a concentration of 3.5μg/ml and Alexa fluor 555-conjugated goat-anti-mouse antibody (Life technologies) diluted 1:1000 was used to detect bound monoclonal antibody on the slide.
